# Editorial: Advancements and challenges in patient centered health systems

**DOI:** 10.3389/frhs.2025.1718153

**Published:** 2025-11-12

**Authors:** Euan Sadler, Anne Rogers

**Affiliations:** School of Health Sciences, University of Southampton, Southampton, United Kingdom

**Keywords:** patient-centred, health systems, barriers & facilitative factors, policy, informal care

Recent developments in health services research have increasingly focused on the evolving landscape of patient-centered health systems (PCHS). This shift reflects growing interest in self-management, informal and alternative sources of care, and expanded access to primary and secondary care. These changes are being accelerated by the rise of the internet and new technologies, particularly artificial intelligence, which promise to deliver expert knowledge directly to patients. These developments challenge and complement traditional, professionally led systems of care.

These transformations are unfolding amid broader social trends: consumer dissatisfaction with existing care models, engagement with alternative health and disease management approaches, and heightened autonomy expectations. Patients now demand greater alignment of power, knowledge, and relationships between themselves and health professionals.

The four articles presented in this Research Topic illuminate both the promise and the fragility of patient-centered advances. They highlight progress, but also areas of regression, reminding us that policy aspirations can be easily undermined.

Walker et al., in *GPs as Strangers*, examine the impact of changes in primary care access on long-term condition management. Their findings reveal a troubling loss of relational continuity and trust between patients and professionals. Patients report feeling excluded and burdened by the process of navigating care. As primary care undergoes redesign, preserving trusted relationships must be central to reform.

Paccagnella et al. explore the effects of COVID-19 restrictions on dementia care in supportive living environments. Their work shows that interruptions to supportive care exacerbate behavioral distress, underscoring the need to safeguard low-risk activities during crises. This highlights how sudden systemic changes can derail patient-centered initiatives.

Morrow et al. review the role of home support workers, whose contributions are critical yet precarious due to low pay, poor working conditions, and limited career progression. The authors call for structured career pathways and investment in digital skills training—essential steps to sustaining this workforce and, by extension, patient-centered care.

Finally, Rivas et al. present findings from the CICADA project, which sought to develop person-centered practices for underrepresented groups. Participatory methods proved effective in fostering authentic engagement; however, persistent challenges—notably inadequate and inconsistent funding—limit sustainability and equity in participation.

Collectively, these contributions convey a clear message: while the rhetoric of advancing patient-centered care remains strong, actual progress is fragile. System redesigns that fail to prioritize patient-centered approaches, alongside crises and shifting policy priorities, risk eroding gains. The path to truly sustainable patient-centered systems requires deliberate investment in relationships, workforce support, and resilience against systemic shocks. Without these foundations, patient-centered care will remain an aspiration rather than a sustained reality.

